# Allergy to cooked, but not raw, peas: a case series and review

**DOI:** 10.1186/s13223-015-0077-x

**Published:** 2015-03-15

**Authors:** Elissa M Abrams, Thomas V Gerstner

**Affiliations:** Section of Allergy and Clinical Immunology, Department of Pediatrics and Child Health, University of Manitoba, FE125-685 William Avenue, Winnipeg, R3A 1S1 Canada

**Keywords:** Food allergy, Pea, Legume

## Abstract

Allergic reactions to legumes are common.Food allergy to cooked, but not raw, pea has been rarely reported in the literature. This case series describes five children who had various IgE-mediated symptoms upon consumption of cooked pea, but tolerated raw pea. Skin testing then confirmed positive responses to cooked, but not raw, peas. It is important to consider allergy to cooked legumes, even in the context of raw legume tolerance.

## Background

Allergic reactions to the legume family are common, and comprehensive reviews have identified the major allergens and cross-reactivity within this family [1]. The major legumes include peas, beans, lupin, lentils, peanut and soy. Prevalence of legume allergy varies by location and type of legume. In Spain, where legumes are consumed frequently and early, legumes were reported to be the fifth most common cause of food allergy in young children [2]. In India, chickpea is a major food allergen [3]. Lupin allergy varied from 4.1% in the Mediterranean to 1.6% in Europe [4,5]. It has been hypothesized that high consumption of legumes may be responsible for increasing sensitization [1]. Legume cross-reactivity also varies by region - while extensive cross-reactivity among lentil, chickpea and pea were reported in the Mediterranean area, only minimal cross-reactivity among legumes (mainly reported between peanut and soy) have been noted in North America [6,7].

There have been two allergens identified in pea (Pis s1 and Pis s2) [8]. Pis s 1 belongs to the vicilim family while Pis s 2 belongs to the convicilin family [1]. However, very few studies have evaluated pea allergy. One recent study described 54 Mediterranean children with legume allergy, of whom 50% were pea allergic. In this population, 74% of pea allergic children were pollen sensitized and the majority were cross-sensitized to other legumes. Of note, this study did suggest that legume allergic children had skin test positivity to boiled extracts more often than raw extracts [9], but did not report clinical allergy to boiled, but not raw, pea. A recent comprehensive review was done on legume allergy [1]. While this review commented on heating effects on various legumes (such as roasting versus boiling peanut), it did not comment at all on how cooking would affect pea reactivity [1]. There has been an article on thermal processing of legume allergens that suggests that, in fact, thermal processing (such as boiling or roasting) decreases IgE-binding capacity for legumes, including peas [10], chickpeas and lentils [11].

Our case series describes a set of patients who clinically reacted to, and were skin test positive to, cooked, but not raw pea. To our knowledge, this is the first case series reporting clinical reactivity to cooked, but not raw, pea.

## Case series

The first patient was a 10 year old female with a history of peanut allergy, and milk allergy (resolved). Upon ingestion of soup containing cooked yellow peas (which she had previously tolerated) two years ago she developed throat pruritus and abdominal pain. There was no associated vomiting, diarrhea, respiratory symptoms, angioedema or urticaria. She was taken to the Emergency Room where no interventions were required. Epinephrine was not administered. Despite this reaction she continued to tolerate fresh peas with no reaction. Skin testing on allergy assessment was positive to boiled peas (maximal wheal diameter of 6 mm) and pea soup (8 mm) but negative to raw pea (2 mm). Specific IgE (done soon after the reaction) was positive to pea (41.2 kU/L). Environmental testing done four years prior to the reaction was uniformly negative (including to tree). She already had an epinephrine auto injector and continues to avoid peanut and cooked peas. She tolerates beans and soy with no reaction.

The second patient was an 11 year old male with a history of kiwi allergy and allergic rhinitis (sensitized to weed). At age four he ingested cooked peas and developed urticaria and throat pruritus. Several years later, he ingested cooked peas again and developed throat pruritus and tongue angioedema with no associated urticaria, vomiting, diarrhea or respiratory symptoms. However, he tolerated fresh peas with no reaction. He tolerates other legumes (peanut, soy, bean). Skin testing was positive to boiled pea (18 mm) but negative to raw pea(2 mm). Specific IgE to pea was 1.62 kU/L. He has avoided cooked pea since this reaction five years ago and carries an epinephrine autoinjector.

The third patient was an 11 year old female with a history of chronic atopic dermatitis, asthma (on Singulair every fall), and allergic rhinitis (sensitized to alternaria, aspergillus, cat and dog). She had a history of egg and peanut allergy (tolerating egg in baked goods only). Three years ago, upon ingestion of cooked pea she developed pruritus of the throat and tongue, with no associated urticaria, angioedema, vomiting, diarrhea or respiratory symptoms. No interventions were required and the reaction resolved quickly. She tolerates fresh snow pea. Except for peanut, she tolerates other legumes ( such as soy and bean). Skin testing to boiled pea was positive (6 mm) but negative to raw pea. Specific IgE to pea (done 3 years after the initial reaction, this year) was negative (<.35 kU/L). She carries an epinephrine auto injector due to her pea, egg and peanut allergy. There have been no accidental exposures to pea and she continues to avoid cooked pea.

The fourth patient was an 11 year old male with a history of asthma and rhinoconjunctivitis (sensitized to cat and cladosporium; on seasonal intranasal steroids and Ventolin PRN), as well as atopic dermatitis. He had a history of peanut, tree nut (cashew, pistachio) and fish allergy. He developed throat pruritus (no respiratory symptoms, urticaria, or diarrhea) soon after ingesting cooked peas two years ago. Reactine was given (no epinephrine) and the reaction resolved within a few hours. He tolerates fresh garden peas with no reaction. He has a history of emesis and lip angioedema with lentils but tolerates beans (confirmed with oral challenge) and soy with no reaction. Skin testing on allergy assessment was positive to boiled peas (10 mm) but negative to raw pea. Skin testing was also positive to fresh lentil (16 mm). He carries an epinephrine auto injector and continues to avoid lentils, cooked peas, as well as peanut, tree nuts and fish.

The fifth patient was a 13 year old male who developed throat clearing and pruritus, scalp pruritus, and facial erythema with pea soup two years ago that self-resolved within an hour. There was no associated vomiting, diarrhea or respiratory symptoms. He ate fresh peas from the garden without a reaction. He also tolerates soy, and beans. There was a history of a similar reaction to peanut three years prior to the pea reaction, and he had been avoiding peanut since. Skin testing was positive to boiled pea (9 mm) but negative to raw pea. Skin testing to fresh peanut was borderline (2 mm). Specific IgE to peanut was 0.4 kU/L but was not done to pea. Environmental testing done at time of assessment was completely negative (including to birch). He has avoided cooked peas and peanut since assessment, and carries an epinephrine autoinjector.

## Conclusions

It is known that heating can alter allergenicity of foods. In the legume family, it has been demonstrated that roasting peanut increases allergenicity [12] while boiling decreases allergenicity [13]. For soy, boiling decreases allergenicity [14]. Heating can also affect diagnostic value of allergy testing. In a study on lentil extracts, it was found that boiled lentil extracts had greater diagnostic value than raw lentil extracts [15]. In a recent review of legume allergy, sensitivity, specificity, and both positive and negative predictive value were greater with boiled lentil, chickpea and pea extract than with raw [9]. It has been shown in the literature that allergenicity of pea is affected by level of seed maturation [16] and fermentation [17]. Notably, a few studies have actually documented lower rate of IgE-binding/allergenicity with heated versus raw legumes [10,11].

It is known that heating can alter the three dimensional structure of proteins, and that in fact different temperature treatments can cause differences to protein structure. For example, it has been shown that at a temperature of 70–80 degrees Celcius, there is a loss of secondary structure while at higher temperatures formation of new bonds and aggregate formation can occur [18].

To our knowledge, this is the first case series reporting clinical reactivity to cooked, but not raw, pea. Although IgE-mediated, the mechanism of epitope formation is not known. It could be hypothesized that heating increases allergenicity through an alteration of protein structure - whether it be stabilization of bonds, neonatigen formation, or alteration of digestibility.

The majority of our patients had a systemic reaction. Of note, two of the five patients documented in the case series had isolated oropharyngeal symptoms, which initially may appear more consistent with oral allergy syndrome. It is not known why these two patients had isolated oropharyngeal symptoms, but reacting to cooked but not raw pea is contrary to oral allergy syndrome, where the reaction should be to the raw protein. In addition, environmental testing was done on both of the patients with isolated oropharyngeal symptoms and neither were pollen allergic at the time of testing.

Component testing is emerging in food allergy, and it is hoped that it will better characterize various food allergy phenotypes, as well as identify patients at risk for anaphylaxis versus oral allergy syndrome. Components for peanut, some tree nuts and soybean have been isolated that indicate likelihood of anaphylaxis risk [19]. To our knowledge component testing has not yet been described for pea allergy, but this emerging field may help further define pea allergy.

Food allergy is becoming more and more prevalent in North America. IgE-mediated reactions to legumes are well described in the literature, but there is a paucity of literature about pea allergy specifically. To our knowledge, this is the first case series describing allergic reactions to cooked, but not raw, pea. One should always consider pea allergy, even in the context of raw pea tolerance. If diagnosed, as with all food allergy, these patients should carry an epinephrine auto injector and be regularly re-assessed.

## Consent

Consent has been obtained by all families described in this case series.
